# The Role of [18F]FDG PET Imaging for the Assessment of Pulmonary Lymphangitic Carcinomatosis: A Comprehensive Narrative Literature Review

**DOI:** 10.3390/diagnostics15131626

**Published:** 2025-06-26

**Authors:** Francesco Dondi, Pietro Bellini, Michela Cossandi, Luca Camoni, Roberto Rinaldi, Gian Luca Viganò, Francesco Bertagna

**Affiliations:** 1 Department of Medical and Surgical Specialties, Radiological Sciences and Public Health, University of Brescia, 25123 Brescia, Italy; francesco.bertagna@unibs.it; 2 Nuclear Medicine, ASST Spedali Civili di Brescia, 25123 Brescia, Italy; pietro.bellini@asst-spedalicivili.it (P.B.); michela.cossandi@asst-spedalicivili.it (M.C.); roberto.rinaldi@asst-spedalicivili.it (R.R.); 3 Clinical Engineering, ASST Spedali Civili di Brescia, 25123 Brescia, Italy; gianluca.vigano@asst-spedalicivili.it

**Keywords:** pulmonary lymphangitic carcinomatosis, PLC, lymphangitic carcinomatosis positron emission tomography, PET/CT, PET, [18F]FDG, FDG, fluorodeoxyglucose

## Abstract

**Background/Objectives**: Pulmonary lymphangitic carcinomatosis (PLC) is a rare, aggressive manifestation of metastatic cancer characterized by lymphatic infiltration of the lungs, typically indicating advanced disease and poor prognosis. **Methods**: This comprehensive narrative review evaluates the role of [18F]fluorodeoxyglucose ([18F]FDG) positron emission tomography (PET) imaging in assessing PLC. **Results**: Current evidence demonstrates that [18F]FDG PET/CT achieves high diagnostic accuracy, with sensitivity and specificity ranging from 86 to 97% and 84 to 100%, respectively, particularly when employing semiquantitative metrics such as peritumoral standardized uptake value (SUVmax) thresholds (e.g., ≥2.1). PET/CT surpasses high-resolution computed tomography (HRCT) in distinguishing PLC from mimics like pulmonary sarcoidosis by identifying distinct metabolic patterns: bronchovascular hypermetabolism in PLC versus subpleural nodular uptake in sarcoidosis. Prognostically, metabolic tumor burden (e.g., SUVmax × involved lobes) and novel cPLC classifications (localized to the ipsilateral or contralateral lung) independently predict progression-free survival. However, challenges persist, including non-specific tracer uptake in inflammatory conditions and variability in SUV measurements due to technical factors. Emerging digital PET/CT systems, with enhanced spatial resolution, may improve the detection of focal PLC and reduce false negatives. While [18F]FDG PET/CT is invaluable for whole-body staging, therapeutic monitoring and biopsy guidance, the standardization of protocols and multicenter validation of prognostic models are critical for clinical integration. Future research should explore novel tracers (e.g., PSMA for prostate cancer-related PLC) and machine learning approaches to refine diagnostic and prognostic accuracy. **Conclusions**: This review underscores the role and the transformative potential of [18F]FDG PET/CT in PLC management while advocating for rigorous standardization to maximize its clinical utility.

## 1. Introduction

Lymphangitis carcinomatosis (LC) is a pathological condition characterized by the presence of malignant infiltration and inflammation of lymphatic vessels due the metastatic spread of a neoplasm affecting a primary site [1,2]. Even though LC can theoretically involve all the organs, it almost always occurs in the pulmonary interstitial lymphatics and is named pulmonary lymphangitic carcinomatosis (PLC). Limited reports of skin, duodenum and kidney LC have also been described [1,3,4,5]. PLC is quite a rare manifestation of the pulmonary spread of a neoplasm, since only about 6–8% of pulmonary metastases are caused by this condition [1].

Different neoplasms can be associated with PLC but its commonest causes include breast, lung, stomach, prostate, pancreas, colon, cervix and uterine malignancies. Less frequent sites of primary tumors have been reported and include the lips, tonsils, pharynx, hypopharynx, mandible, placenta, kidneys, liver, skin, sarcoma and unknown primary sites [1,6,7,8,9,10,11,12,13,14,15,16,17,18,19]. In general, the presence of PLC is related to poor life expectancy as it usually represents an end-stage manifestation of malignancies and its extension can also influence the prognosis of these patients [1,6,20,21,22,23,24,25].

The clinical presentation of PLC is mainly related to the presence of progressive dyspnea which is usually the most common symptom in almost 60% of the subjects. In addition, pleuritic chest pain may be present if the neoplasm obstructs the subpleural lymphatics. Dry cough can also be developed by these patients. More uncommon presentations include hemoptysis, weight loss, lethargy, low-grade fever, tachypnoea and tachycardia. Interestingly, asymptomatic cases have also been described in the literature [1,6,26,27]. Approximately half of the patients with PLC will die within 3 months after the onset of respiratory symptoms [28,29].

From a diagnostic point of view, the suspicion of the presence of PLC is often related to the clinical background with the support of patient history, clinical features and imaging findings. Laboratory findings and bronchoalveolar lavage could moreover add particular information that can help in the final diagnosis [1,30,31]. However, biopsy and histopathological confirmation are mandatory to provide a definite confirmation of the presence of PLC [6]. While biopsy is the gold standard for PLC diagnosis, practical challenges exist. Diffuse lymphatic infiltration often complicates targeted sampling, risking false negatives. Additionally, procedural risks in critically ill patients may limit feasibility, emphasizing the role of non-invasive imaging for initial evaluation and biopsy guidance. Imaging plays a fundamental role in the assessment of this disease, even though it is often performed to diagnose or rule out other causes of dyspnea or cardio-respiratory symptoms, rather than to specifically evaluate PLC [1]. The exams that are usually employed for its evaluation encompass conventional imaging modalities, such as chest X-ray and computed tomography (CT), since they can demonstrate the presence of specific patterns and features that can help in the final diagnosis, such as extensive peribronchovascular consolidation [1,32,33,34]. Lung ultrasound can also give some information on PLC characteristics [35]. Treatment strategies for PLC rely on surgical resection, chemotherapy, radiotherapy and/or a combination of these therapies, and their success is related to the response of the primary neoplasm that causes PLC [1,28,36,37,38,39,40,41,42,43]. Moreover, corticosteroids are recommended by the clinical guidelines [44]. However, the diagnosis of PLC is considered a feature of end-stage malignancies and therefore, as mentioned, the life expectancy of these subjects is poor, with different reports ranging from several days to 3 years [1,6,28,45,46].

In the past, different nuclear medicine imaging techniques have been used to evaluate PLC, with various results [47,48,49,50,51]. In recent years however, positron emission tomography (PET) imaging has demonstrated its ability to assess different neoplastic conditions since different radiotracers, which are able to image several metabolic pathways or various receptor expressions, have been produced and proposed [52]. In particular, [18F]fluorodeoxyglucose ([18F]FDG) is the most used and available radiopharmaceutical worldwide, since it has the ability to assess the glycolytic activity of cells in several pathological conditions, both neoplastic and benign [53]. In particular, [18F]FDG can enter the cells using glucose transporter 1 (GLUT1) and is therefore phosphorylated by hexokinase to [18F]FDG-6 phosphate ([18F]FDG-6P). Unlike glucose-6 phosphate (G-6P), [18F]FDG-6P cannot be further metabolized because it lacks the 2-hydroxyl group (replaced by [18F]) needed for glycolysis, so it becomes trapped into the cell. As a consequence, [18F]FDG is used to assess the glucose uptake of cells [54,55,56]. In the thoracic district [18F]FDG PET imaging has proven its ability to aid in the diagnosis, staging and evaluation of responses to therapy, and the prognostication of different neoplastic conditions, such as lung cancer and primary mediastinal lymphoma [57,58,59,60].

The aim of this comprehensive review is to summarize the available literature and evidence on the role of [18F]FDG PET imaging for the assessment of PLC.

## 2. Methods

A literature search to find relevant papers assessing the role of [18F]FDG PET imaging in PLC was performed on different databases, such as PubMed/MEDLINE, Scopus and Embase. The search had no beginning date limit and was updated until 1 May 2025. Only articles published in the English language were considered. Preclinical studies, conference proceedings, reviews and editorials were excluded. The references of the retrieved articles were also screened for additional papers to expand the final database.

## 3. The Role of [18F]FDG PET Imaging for the Evaluation of PLC

### 3.1. First Reports

Several reports have indicated that [18F]FDG PET imaging is able to reveal the presence of PLC in different malignancies [32,61,62,63,64,65,66]. First, it was reported that diffuse increased tracer uptake in the bilateral lung along thickened bronchovascular bundles in the presence of PLC was related to breast cancer spread, with a standardized uptake value (SUV) of 5.2 and an SUV ratio between the mediastinal blood pool and PLC of 0.44 [61]. Tracer uptake along thickened bronchovascular bundles and bilaterally scattered ground glass opacities were also present in a case of PLC related to colon cancer [32].

Diffuse tracer uptake related to the presence of lung adenocarcinoma-derived PLC has been reported, with an SUVmax of 3.4 in the presence of typical CT alterations [65]. Additionally, reports on the value of [18F]FDG PET imaging to demonstrate a positive or negative response to chemotherapy have been published [63,65]. Moreover, a single report revealed the possible ability of this imaging modality to define PLC as the cause of chronic cough in the case of a negative chest high-resolution CT (HRCT), further revealing the underlying primary neoplasm in the colon [62]. Moreover, in addition to PLC, other metastatic sites related to the same primary tumor may be revealed in the same PET scan [32,62,66].

Even though [18F]FDG is a tracer not usually used for the assessment of prostate cancer (PCa), a report revealing intense hypermetabolic activity in the lungs related to PLC derived from this neoplasm has been proposed [66]. Interestingly, focusing on PCa, a comparison of PLC appearance on [18F]FDG and [18F]-DCFPyL PET imaging has been published, revealing diffuse tracer uptake in both cases; however, partial overlap between them was demonstrated when assessing bone metastases. Moreover, serial PET/CT scans may also be informative in evaluating the response to therapy in this neoplasm [64].

### 3.2. Clinical Studies

As recently reported, [18F]FDG PET imaging is able to evaluate the presence of PLC by demonstrating intense and diffuse tracer uptake along typical bronchovascular bundle alterations. On these bases, some clinical studies assessing the role of this imaging modality more precisely have therefore been produced [67,68,69,70,71,72] (Table 1 and Table 2).

First, Digumarthy et al. [67] confirmed the presence of diffuse increased uptake in seven patients with PLC areas previously demonstrated with CT scans, with a mean SUV value of 1.91 ± 0.78 (range: 1.1–3.5), a value significantly higher than the background SUV of the normal contralateral lung (*p* = 0.003). Furthermore, the mean ratio between the SUV of the blood pool and the SUV of PLC was 0.86 ± 0.36 (range: 0.5–1.6), again significantly higher than the ratio with the normal contralateral lung (*p* = 0.0002). The ratio of the SUV PLC lung to the corresponding normal contralateral lung was significantly increased (*p* = 0.006). Based on their findings, the authors proposed that the presence of diffuse tumor deposition in the interstitium was a feature typical of PLC and was able to accumulate [18F]FDG, therefore resulting in the increased radiopharmaceutical uptake and higher SUV of these areas.

Acikgoz et al. [68] usefully described different spectrums of [18F]FDG uptake related to the presence of PLC in five different subjects. In particular, diffuse increased uptake involving whole lungs and/or the pleura or only a limited part could be present. Similarly to CT, the degree of the pathological process of PLC could be extensive or limited: diffuse, lobar or segmental tracer uptake was present in extensive cases, while a hazy area of uptake or linear uptake extending from the tumor to the lymph nodes could be seen in limited PLC. Interestingly, the nature of the dissemination of the disease, as demonstrated by the tracer uptake, could influence the therapeutic management and therefore the prognosis of these patients. Additionally, this imaging modality was also able to highlight the presence of the primary tumor and/or the presence of its metastatic spread. Curiously, PLC could also be characterized by the presence of lower-grade uptake when compared to the primary underlying lesion. Based on the [18F]FDG PET findings, the authors proposed a possible interpretation of lymphangitic spread through the seeding of tumor cells to the bronchovascular lymphatics, the direct invasion of lymphatics from the lung tumors or through retrograde lymphatic invasion from a hilar lymph node.

Prakash et al. [69] focused their attention on 35 PLC patients, reporting that 30 of them demonstrated [18F]FDG uptake on the areas involved by the disease, therefore revealing a sensitivity of 86%. Interestingly 4/5 of the patients without uptake had focal PLC while the other had a diffuse pattern. Subjects with PLC missed by [18F]FDG PET imaging had a primary lung lesion (4 patients) or metastatic nodules (1 patient) close to the areas involved by PLC, therefore making the distinction between the primary lesion and the lymphatic involvement difficult. Since no abnormal uptake was present in normal tissues, the specificity of PET imaging was 100%. Moreover, increased [18F]FDG uptake was demonstrated in hilar and mediastinal lymph nodes in 19 patients. The mean SUV in PLC areas was significantly higher than in the contralateral lung (*p* < 0.001). The mean SUV in the lung with PLC and in the contralateral lung were 1.37 ± 0.64 (range 0.5–2.9) and 0.51 ± 0.29 (range 0.1–0.8), respectively. The ratio between the SUV of the mediastinal blood pool and lymphangitic lung was significantly higher than the same ratio for the normal contralateral lung (*p* < 0.0001).

More recently, Jreige et al. [70] evaluated the performances of HRCT and [18F]FDG PET/CT for the diagnosis of PLC by comparing subjects with (69 patients) and without this pathology. For HRCT, among the different features, only the presence of peribronchovascular thickening was significantly correlated with the presence of PLC (odds ratio [OR] 10.95; 95% confidence interval [CI], 3.33–36.0; *p* < 0.001), showing an area under the curve (AUC) of the receiver operating characteristic (ROC) analysis of 0.76 (95% CI, 0.67–0.85). Its sensitivity, specificity and positive and negative likelihood ratios were 69%, 83%, 4.12 and 0.38, respectively. Focusing on PET/CT, the ability of qualitative assessments of peritumoral increased uptake, SUVmax, SUVmean, metabolic tumor volume (MTV), total lesion glycolysis (TLG), peritumoral SUVmax, peritumoral SUVmean, peritumoral SUVmax ratio with contralateral lung and peritumoral SUVmean ratio with contralateral lung were evaluated, and all of them were significantly different between patients with PLC and subjects without this pathology (*p* < 0.001 for all of them). Qualitative evaluation of peritumoral uptake performed similarly to peribronchovascular thickening, with AUC, sensitivity and specificity of 0.89 (95% CI, 0.81–0.97), 94% and 84%, respectively (*p* = 0.054). Focusing on semiquantitative PET/CT analysis, interobserver reproducibility of peritumoral SUV measurements were excellent. Peritumoral SUVmax and SUVmean were highly associated with PLC with ORs significantly higher than the ORs for qualitative peritumoral evaluation (*p* = 0.0022 and 0.0005, respectively) and than the ORs for peribronchovascular thickening (*p* = 0.0004 and 0.0001, respectively). A cutoff value of 2.1 for peritumoral SUVmax had the best diagnostic performance, with an AUC of 0.98 (95%CI, 0.96–1.00), a sensitivity of 97% and a specificity of 92%, higher than the qualitative assessments of peritumoral uptake or peribronchovascular thickening (*p* = 0.0064 and <0.0001, respectively). Similar findings were demonstrated with a cutoff of 1.2 for SUVmean. Moreover, absolute peritumoral SUVmax, peritumoral SUVmean, peritumoral SUVmax ratio and peritumoral SUVmean ratio had similar diagnostic performances.

Ji et al. [71] performed an interesting study aimed to distinguish pulmonary sarcoidosis (PS) and PLC (58 patients) on the basis of [18F]FDG PET/CT findings. In terms of nodal involvement, patients with PS had significantly higher areas of lymphatic involvement, incidences of bilateral hilar involvement, shorter diameters of lymph nodes and lower average SUVmax compared to PLC subjects (*p* < 0.01). In the case of pulmonary features, PS patients had lower incidences of bronchovascular bundle thickening, pleural thickening, interlobular septal thickening, centrilobular peribronchovascular interstitial thickening and pleural effusion, while the number of involved lobes, the incidence of large paravascular solitary nodules and the average SUVmax of lung lesions was lower in the PLC group (*p* < 0.05). Moreover, regarding the interstitial thickening pattern, nodular thickening was predominant in the PS group, while diffuse thickening was more evident in PLC patients. Focusing on metabolic pattern, subpleural hypermetabolic activity was prevalent in PS subjects while bronchovascular bundle hypermetabolic activity and lobar diffuse hypermetabolic activity were more common in the PLC group. The authors proposed a diagnostic model with five imaging features selected on the basis of stability and fitting effect: area of lymph node involvement, bronchovascular bundle diffuse thickening, interlobular septal thickening, pleural effusion and subpleural hypermetabolic activity. The AUC of this model in differentiating between PS and PLC was 0.973 (95% CI 0.925–0.994), with sensitivity, specificity and positive and negative likelihood ratios of 87.50%, 98.28%, 50.75 and 0.13, respectively.

Lastly, Park et al. [72] evaluated the prognostic role of pretherapeutic [18F]FDG PET/CT imaging in 50 patients with non-small cell lung cancer (NSCLC)-derived PLC. The median primary tumor size and SUVmax were 43.50 mm (range of 12–69 mm) and 11.15 (range of 3.47–32.00), respectively. A new three-grade system (cPLC) to classify PLC extension was proposed: lymphangitis confined to the lobe of the primary tumor (cPLC1), lymphangitis in other ipsilateral lobes (cPLC2) and lymphangitis affecting the contralateral lung (cPLC3). The location and number of lobes involved by PTC were the same when comparing HRCT and PET/CT findings. The median PLC SUVmax and metabolic PLC burden (the product of PLC SUVmax and number of lobes with PLC) were 2.03 (range of 1.05–8.39) and 2.16 (range of 1.05–21.00), respectively. In the univariate analysis, clinical stage, treatment modality, cPLC, primary tumor SUVmax and metabolic PLC burden were significant prognosticators for progression-free survival (PFS). The optimal cut-off values for PLC SUVmax, primary tumor SUVmax, metabolic PLC burden and age were 1.59, 6.86, 8.39 and 69, respectively. After several adjustments to control for confounding effects, three predictive models for PFS were built: Model 1 was composed of metabolic PLC burden, primary tumor SUVmax and treatment modality; Model 2 consisted of cPLC, primary tumor SUVmax and treatment modality; and Model 3 was composed of clinical stage, primary tumor SUVmax and treatment modality. In the multivariate analyses of the three models, metabolic PLC burden, cPLC and clinical stage were independent prognosticators for PFS. Interestingly, significant differences in prognosis between cPLC1 and cPLC3 and between cPLC2 and cPLC3 were demonstrated (*p* = 0.0002 and 0.0307, respectively). Focusing on overall survival (OS), no [18F]FDG PET parameters revealed a prognostic ability.

## 4. Discussion

In general, the studies included in the review underlined a possible role for [18F]FDG PET imaging to assess the presence of PLC; its main performance in diagnosing and managing PLC are presented in Table 3.

Different reports have suggested that PLC is visualized by an intense diffuse uptake of tracer in the pulmonary districts that are involved by the disease, usually represented on CT images as thickened bronchovascular bundles [32,61,67]. Interestingly, in the case of extensive PLC, it has been proposed that diffuse, lobar or segmental tracer uptake is present, while a hazy area of uptake or linear uptake extending from the tumor to the lymph nodes can characterize limited forms [68]. The paper by Acikgoz et al. [68] could be used as an interesting and useful pictorial guide to the evaluation of PLC with [18F]FDG imaging. Possible interpretations of PLC spreading derived from PET imaging have been proposed, including the lymphangitic spread through the seeding of tumor cells to the bronchovascular lymphatics, the direct invasion of lymphatics from lung tumors or through retrograde lymphatic invasion from a hilar lymph. Curiously, PLC also demonstrated different degrees and lower-grade uptake when compared to the primary underlying neoplasm [68].

One of the main points that is necessary to underline is the fact that the previously highlighted PET patterns related to the presence of PLC are not specific to this condition. For example, diffuse pulmonary [18F]FDG uptake can be observed in the presence of pulmonary vein congestion or stenosis, after radiation treatment, infection or malignant pleural effusion [68,73,74]. More particularly, tracer uptake can also be seen on other interstitial lung diseases such as interstitial pneumonia, PS and cancer-associated sarcoid-like reactions [32,75]. Interestingly, a false finding of PLC related to the presence of pembrolizumab-associated pneumonitis has also been reported [76]. In this setting, the knowledge of the patient’s clinical history and the temporal relationship of imaging manifestation to the phase of the disease, in addition to the methods of treatment, are fundamental pieces of information that can be useful to differentiate PLC from conditions with a similar appearance.

Differences in the manifestation of PS and PLC on PET/CT have been reported: the first condition mostly exhibits subpleural metabolic activity, while the second is mainly characterized by increased metabolism in the bronchovascular bundles and diffuse lobes. A diagnostic model, including subpleural hypermetabolic activity as a variable, with high clinical value in differentiating these two diseases has also been built. Nevertheless, it is mandatory to highlight that even though PS had a higher SUVmax compared to PLC, this parameter has a limited value in the differentiation between these two conditions since a high overlap between them was present [71].

As mentioned, cases of false negative PLC findings have been described and the reasons for this oversight have been related to the limited spatial resolution of PET scanners and the blooming of [18F]FDG uptake in the primary neoplasm that can therefore hide the less intense uptake of PLC [69,76]. New digital PET/CT tomographs, characterized by higher spatial resolutions, will hopefully reduce these issues [77]. In this setting, the improvements of this new technology deriving from an improved image quality have been proposed in different clinical scenarios, in particular for lesion detectability of small-sized lesions. Specific comparison of classic analog and digital PET scanners in the specific clinical setting of PLC have not yet been reported, but the introduction of the last type of scanner will probably help to better define and characterize PLC lesions, therefore increasing the confidence of nuclear medicine physicians.

Not only has the qualitative evaluation of PET scans been proposed as useful to image PLC but also its semiquantitative assessment. In particular, SUV has been used and proposed in different settings to evaluate neoplastic conditions affecting the lung, revealing, for example, its value in prognostic and therapeutic settings [78,79]. In this review, different SUVmax values have been reported for PLC, ranging from 1.37 to 9.1, and similarly, different PLC-to-blood pool ratios, ranging from 0.44 to 3.2, have been proposed; in all the cases, the SUVmax values of the regions affected by PLC were higher than the values of the normal lung [61,65,67,69,70]. Interestingly, it has also been reported that semiquantitative PET parameters performed better than qualitative analysis, while qualitative evaluation and HRCT had similar performances [70]. In this scenario, it is mandatory to highlight that different factors can influence the quantification and interpretation of PET scans [80]. As a consequence, even though these encouraging findings may suggest a clinical role for semiquantitative PET evaluation, the high variability of the reported values across different studies have to be researched in the fact that different tomographs and different acquisition and reconstruction methods have been used among the different papers. Overall, this does not allow us to generalize these findings and to suggest a single SUV value to endorse the presence of PLC.

As mentioned, CT is a fundamental tool for the assessment of pathological conditions affecting the lung, such as PLC. PLC is typically characterized by the presence of interstitial and peribronchovascular thickening, consolidations and nodules, usually bilateral [81]. Moreover, common findings on HRCT include the thickening of interlobular septa and the peribronchovascular interstitium, subpleural nodules, the thickening of the interlobar fissures, pleural effusion, pleural carcinomatosis and hilar and mediastinal nodal enlargement, with relatively little destruction of the overall lung architecture. However, smooth or thickened interlobular septa, in particular if characterized by a nodular appearance, are not specific for PLC and can also be encountered in other interstitial disorders, such as PS. Additionally, hilar adenopathies and effusions have also been reported in the presence of PLC [70,82,83,84]. Most of the studies and the reports included in the present review were performed by using hybrid PET/CT tomographs, with a few exceptions [67,68]. Fused PET/CT imaging are nowadays the gold standard for the acquisition and the interpretation of metabolic imaging, allowing a combination of both functional and anatomical information [85]. The integration of simultaneously acquired morphologic CT and functional PET data is superior to separate imaging examinations, as it enables sampling of the correct area of PLC by the fusion of information from both modalities. Moreover, it can avoid pitfalls due to interval development of confounding factors between the PET and CT examinations, such as, for example, infection [69]. Interestingly, it has been reported that in the specific setting of PLC, the combination of this information can provide insight into the overall tumor burden of the disease and may therefore be useful to predict the prognosis of these patients [72].

One of the added values of [18F]FDG PET imaging is the fact that this is a total body technique, which is therefore able to eventually indicate the primary underlying neoplasm responsible for PLC or to underline the presence of metastatic spreading to other tissues and organs, therefore resulting in the clear staging of the tumor [32,62,64,66,68,75,86]. Furthermore, a definitive histopathologic diagnosis of PLC and/or the primary neoplasm is usually required; therefore, PET imaging may provide useful guidance in the detection of the most appropriate biopsy site, when required [75,86].

A single paper analyzed the prognostic value of [18F]FDG PET imaging in NSCLC patients with PLC, revealing that the metabolic tumor burden (containing both information on tumor metabolism and extension) and cPLC (representing location and extension [20,24,87,88]) were identified as independent prognosticators for PFS [72]. Moreover, several case reports have underlined that by performing multiple and subsequent PET evaluations in the assessment of response to therapy, this imaging modality can give important information able to restage the disease, therefore tailoring the therapeutic management of these patients [63,64,65]. Despite this, the data presented here are related to small cohorts of patients and need to be better clarified and defined in future research.

Interestingly, a case report comparing [18F]FDG and [18F]-DCFPyL PET imaging in PCa-derived PLC revealed similar findings in both modalities [64]. Prostate-specific membrane antigens (PSMAs) are a group of PET tracers that also include [18F]-DCFPyL and are useful for the evaluation of PCa and other different clinical conditions [89,90]. Several reports have underlined the ability of PSMA PET and single photon emission computed tomography (SPECT) imaging to demonstrate the presence of PLC related to PCa [51,91,92,93]. Interestingly, a report revealed intense [18F]FDG uptake in the presence of PLC in a patient with PCa [66]. Therefore, even though this imaging modality is not usually performed to assess the presence of PCa, except for undifferentiated or particular forms [90], its possible role for the evaluation of PLC needs to be better defined.

Artificial intelligence, machine learning (ML) techniques and radiomics features extracted from PET and/or CT imaging are fields of research that are continuously gaining importance in different clinical applications. For example, in the specific setting of lung pathologies, different reports have underlined the possible role of radiomics analysis applied to diverse imaging modalities to differentiate pulmonary inflammatory diseases from lung cancer [94,95,96,97]. Additionally, the added value of ML and radiomics feature extraction from PET imaging has been demonstrated in different clinical settings in the case of lung cancer, such as, for example, diagnosis or prognosis prediction [59,98,99]. Specific evaluation of the possible role of the application of these techniques in differentiating between PLC and inflammatory diseases or conditions with similar appearance on PET imaging have not yet been reported; therefore, future research on this topic will help to define their role in these settings.

Future directions of PET applications in PLC could be to develop standardized acquisition protocols to minimize SUV variability and the use of SiPM digital PET/CT scanners. Emerging digital PET/CT systems, with improved spatial resolution (1.4–2.0 mm vs. 4–5 mm in analog systems), may enhance PLC detection by reducing partial volume effects. This technology could better distinguish focal PLC adjacent to hypermetabolic primaries and mitigate false negatives, as demonstrated in recent lymphoma studies [77]. As mentioned, a single study reported the prognostic impact of [18F]FDG PET imaging in patients with PLC [72]. These encouraging results must, however, be validated in larger multicentric cohorts in order to achieve a clear clinical significance and therefore allow a precise patient-centered approach. The exploration of novel PET tracers is another point that could be explored in future research; as an example, several cases of PSMA uptake have been underlined in the presence of PLC related to the presence of PCa [64,91,92]. Interestingly, it has also been reported that PLC might express a positivity to [99mTc]-fibroblast activation protein inhibitor (FAPI) [51]; therefore, the corresponding PET tracers should also be evaluated in future studies to strengthen their added value. Again, future directions for the assessment of the role of PET imaging in PLC should also be focused on the application of ML techniques and models to enhance its diagnostic accuracy. Lastly, larger studies validating semiquantitative thresholds are needed.

Several limitations affect the studies and the reports that were mentioned in this paper, and therefore influence the generalizability of what has been reported. First of all, the cohorts of the studies were composed of limited samples, and many of them were simple case reports. Additionally, when focusing on the semiquantitative evaluation of PLC, a high variability of SUV and SUV ratio values between different papers has been reported, a fact that, as mentioned, may be related to the different scanners used [80]. Variability in PET/CT protocols—including differences in scanners, acquisition parameters and SUV calculation methods—may affect the reported diagnostic thresholds and reduce comparability across studies. In addition, it is known that different technical, physical and biological factors are able to influence the quantification of tracer uptake in PET imaging, therefore resulting in possible differences in SUV values [100]. Despite this, it has, however, been reported that when acquired with careful attention to the protocols used, SUV still remains as a highly repeatable metric [101]. In this setting, the need for the standardization of SUV measurements across different centers remains a central point of PET studies that, when achieved, will help to strengthen the findings proposed. Other limitations may be related to the publication bias favoring positive results that could overemphasize the efficacy of [18F]FDG PET/CT, while insufficient long-term follow-up data in prognostic analyses may compromise the validity of survival correlations. Additionally, confounding factors such as inflammation or infection, which mimic PLC’s metabolic activity, are not consistently addressed, potentially inflating specificity estimates. Lastly, one of the significant drawbacks of many studies was the lack of histopathological confirmation of PLC, which remains crucial for its precise diagnosis. As mentioned, different causes of false positive findings may be related to PLCin; therefore, a precise final diagnosis of the PET findings can only be formulated with a clear and definitive histopathological evaluation. However, procedural risks in critically ill patients may limit its feasibility. Again, this fact is desirable in future studies, to strengthen the added value of [18F]FDG imaging in the evaluation of PLC.

## 5. Conclusions

In conclusion, [18F]FDG PET imaging seems to be a reliable tool to assess the presence of PLC. In addition, some insights on its value in different clinical settings, such as, for example, the differential diagnosis with PS or its prognostic ability, have emerged but require validation through future research. Prospective multicenter trials are needed to validate the prognostic utility of the metabolic burden of PLC and cPLC staging. Collaborative efforts to standardize SUV measurements across institutions, coupled with machine learning models integrating PET/CT and clinical data, could refine risk stratification and personalize therapeutic strategies.

## Figures and Tables

**Table 1:** Characteristics of the studies considered for this review.

First Author	N. Ref.	Year	Country	Study Design	N. Pts.	PLC Pts. (%)	Primary Neoplasms
Digumarthy SR	[67]	2005	USA, Singapore	Retrospective	14	7 (50)	Lung
Acikgoz G	[68]	2006	USA	Retrospective	5	5 (100)	Lung, esophagus
Prakash P	[69]	2009	USA	Retrospective	35	35 (100)	Lung, prostrate, breast, pancreas, head and neck, unknown primary
Jreige M	[70]	2020	Switzerland	Retrospective	94	69 (73)	Lung
Ji Y	[71]	2023	China	Retrospective	114	58 (51)	Breast, stomach, cervix, unknown primary, colon, liver, bile duct, duodenum, rectum, kidney, thymus, parotid gland, ovary
Park YJ	[72]	2023	Republic of Korea	Retrospective	50	50 (100)	Lung

N.: number; Pts.: patients; Ref.: reference; USA: United States of America; PLC: pulmonary lymphangitic carcinomatosis.

**Table 2:** Results and main findings of the studies considered for this review.

First Author	Ref.	Device	Reported Mean Activity (MBq)	PET Analysis	Main Findings
Digumarthy SR	[67]	PET	555–740	Qualitative and semiquantitative	Diffuse increased tracer uptake is present on CT pattern of PLC.
Acikgoz G	[68]	PET	370–444	Qualitative and semiquantitative	Typical spectrum and findings of [18F]FDG PET in the presence of PLC are presented.
Prakash P	[69]	PET/CT	555–740	Qualitative and semiquantitative	PET/CT has high specificity (100%) in the detection of PLC; however, it has 86% sensitivity when located close to a primary neoplasm.
Jreige M	[70]	PET/CT	3.7 ± 0.5/kg	Qualitative and semiquantitative	Semiquantitative analysis of [18F]FDG PET/CT outperforms qualitative evaluation and HRCT for the diagnosis of PLC. These last two modalities perform similarly.
Ji Y	[71]	PET/CT	4.4–5.5/kg	Qualitative and semiquantitative	Different manifestations on [18F]FDG PET/CT imaging are present between PLC and pulmonary sarcoidosis.
Park YJ	[72]	PET/CT	5/kg	Qualitative and semiquantitative	[18F]FDG PET/CT gives some prognostic information in NSCLC patients with PLC.

PLC: pulmonary lymphangitic carcinomatosis; [18F]FDG: [18F]fluorodeoxyglucose; PET: positron emission tomography; CT: computed tomography; MBq: megabecquerel; Ref.: reference; kg: kilogram; HRCT: high-resolution CT; NSCLC: non-small cell lung cancer.

**Table 3:** Performance of [18F]FDG PET imaging in PLC.

Increased uptake on PLC localization. High specificity, lower sensitivity when close to lung neoplasm or metastasis. Semiquantitative evaluation enhances the diagnostic accuracy. Whole-body technique. Differential diagnosis between PLC and PS. Prognostic information.

PLC: pulmonary lymphangitic carcinomatosis; [18F]FDG: [18F]fluorodeoxyglucose; PET: positron emission tomography; PS: pulmonary sarcoidosis.

## Data Availability

Data supporting the reported results can be found using the public scientific databases.
